# Validation of the BD FACSPresto system for the measurement of CD4 T-lymphocytes and hemoglobin concentration in HIV-negative and HIV-positive subjects

**DOI:** 10.1038/s41598-020-76549-6

**Published:** 2020-11-11

**Authors:** Xiaofan Lu, Hanxiao Sun, Haicong Li, Wei Xia, Hao Wu, Daihong Chen, Meiyu Tan, Shijun Yu, Tong Zhang, Huiming Sheng, Zhaoqin Zhu

**Affiliations:** 1 Center for Infectious Diseases, Beijing Youan Hospital, Capital Medical University, Beijing, China; 2 Beijing Key Laboratory for HIV/AIDS Research, Beijing, China; 3 Shanghai Tong Ren Hospital, Shanghai Jiao Tong University School of Medicine, Shanghai, China; 4 Shanghai Public Health Clinical Center, Fudan University, Shanghai, China; 5 BD Biosciences, 2350 Qume Drive, San Jose, CA 95131 USA

**Keywords:** Biological techniques, Biotechnology

## Abstract

This study aimed to compare the performance of the BD FACSPresto system with the conventional standard-of-care technologies for the measurement of absolute CD4 count (AbsCD4), CD4 percentage (CD4%) and total hemoglobin concentration (Hb) in capillary and venous blood samples of HIV-negative and HIV-positive subjects. A total of 1304 participants were included in this prospective cohort study. Both venous and capillary blood samples were analyzed using the BD FACSPresto system and the results were compared against the BD FACSCalibur for enumerating AbsCD4 and CD4% and Sysmex XT-4000i hematology analyzer for determining Hb levels. Method comparison studies were performed using Deming regression and Bland–Altman plots. The Deming regression analyses comparing the accuracy of the BD FACSPresto system with the reference standard technologies demonstrated a significant linear correlation between the AbsCD4, CD4%, and Hb values generated by the two platforms. The 95% CI of the slopes for AbsCD4, CD4%, and Hb levels were 0.94–0.99, 0.99–1.01 and 0.86–0.93, respectively (*P* < 0.001). Bland–Altman plots for AbsCD4, CD4%, and Hb levels demonstrated close agreement between the BD FACSPresto system and the reference standards for all study participants. The performance and accuracy of BD FACSPresto system was comparable to the reference standard technologies. The BD FACSPresto system can be used interchangeably with BD FACSCalibur platform for CD4 and Sysmex XT-4000i hematology analyzer for Hb concentrations in resource-limited settings thus, improving accessibility to point-of-care testing services.

## Introduction

According to data from the World Health Organization (WHO), an estimated 38 million people worldwide were living with human immunodeficiency virus (HIV) at the end of 2019, with approximately 68% of adults and 53% of children accessing antiretroviral therapy (ART)^1,2^. Globally, there were 1.7 million new cases of HIV infection in 2019, and around 690,000 deaths were reported due to HIV/acquired immunodeficiency syndrome (AIDS)^1^. The number of people living with HIV in China has increased markedly from around 307,000 in 2010 to 501,000 in 2014^3^, and further to 758,000 in 2017^4^. Furthermore, there were more than 134,000 new cases of HIV/AIDS in 2017 in China, with more than 95% of these infections acquired through sexual transmission, and nearly 31,000 deaths due to HIV/AIDS occurred during the same year^4^. At the end of 2018, it was reported that approximately 1.25 million people were living with HIV in China. Globally, only about 81% of people with HIV knew their HIV status in 2019. The remaining 7.1 million people (approximately 19%) still needed access to HIV testing services. Therefore, increasing access to HIV testing is essential for its management including, prevention, treatment, care and support services.

It is well known that HIV targets cells expressing the CD4 antigen (T-helper cells, macrophages and dendritic cells) by binding to the CD4 molecule via the envelope protein gp120, thus resulting in progressive deterioration of the immune system, which further leads to the development of life-threatening opportunistic infections and cancers^1,5^. Therefore, evaluation of absolute CD4 T lymphocyte counts (AbsCD4) and CD4 percentages (CD4%) have been the mainstay criteria for monitoring HIV disease progression and determining patient prognosis. Moreover, the most common opportunistic infections associated with AIDS usually occurred at a clinically relevant AbsCD4 cutoff of less than 200 cells/μL or a CD4% cutoff of less than 14^6^. Therefore, development of methods to monitor the decline in CD4 count would be extremely important, especially in subjects with co-infections or opportunistic infections. Routine and timely monitoring of the CD4 count in HIV-infected patients aids in the understanding of an individual’s immune status, disease progression, determining appropriate time for treatment initiation, evaluating treatment efficacy, and assessing clinical complications^7^. Patients with an AbsCD4 count of greater than 500 cells/µL after ART are considered to have achieved restoration of immune function^8,9^ and have a life expectancy similar to that of the general population^10^. Moreover, CD4 cell count is a reliable indicator for initiating ART in the absence of a viral load measurement^11,12^.

Anemia is the most common hematologic abnormality affecting HIV-infected individuals. The prevalence of anemia among HIV-positive patients in China ranges from 39.2% to 51.9%^13,14^. Moreover, the incidence of anemia is strongly associated with poorer prognosis, independent of CD4 count and viral load^15,16^. In addition, although highly active ART (HAART) often improves anemia in patients with HIV^17–19^, drugs such as zidovudine can induce anemia as an adverse effect of treatment^20,21^. Therefore, monitoring hemoglobin (Hb) levels is not only important in the assessment of disease progression and response to therapy, but also in the detection of adverse events caused by HAART.

The emergence of point-of-care (POC) testing in recent years has led to the decentralization of ART in resource-limited settings and further enabled rapid and early screening for HIV, thus increasing the number of individuals who are aware of their HIV status, particularly in low- and middle-income countries^22^. One such POC instrument is the BD FACSPresto system (BD Biosciences, San Jose, CA, USA), which is a portable device that provides measurements of AbsCD4, CD4%, and Hb levels using a single drop of venous or capillary blood^23^. The use of such device that simultaneously determines AbsCD4, CD4% and Hb levels from capillary and/or venous blood would simplify the routine monitoring of patients with HIV/AIDS and be particularly beneficial in resource-constrained settings. The BD FACSPresto system has been validated in HIV-infected patients in Kenya, India, Thailand, and the USA and is commercially available in North America, the European Union, and some African countries^23^. However, no assay kits or instruments are currently approved in China for the simultaneous measurement of AbsCD4, CD4%, and Hb levels in capillary and venous blood samples. Therefore, the aim of this study was to evaluate whether the performance and accuracy of the BD FACSPresto system in the measurement of AbsCD4, CD4%, and Hb levels in capillary and venous blood samples of HIV-negative and HIV-positive patients is comparable to those of commercially available standard-of-care systems.

## Methods

### Study design and participants

This multicentric, prospective cohort study comprised of HIV-infected patients recruited from the Beijing YouAn Hospital and the Shanghai Public Health Clinical Center, and healthy subjects recruited from the Shanghai Tongren Hospital during March–May 2018. The diagnosis of HIV/AIDS was based on the Diagnostic Criteria for HIV/AIDS (WS 293-2008)^24^.

All adults aged 18 years and above including, pregnant women were considered eligible for the study. HIV-positive individuals and pregnant adolescent girls below 18 years of age, and those who declined finger-prick collection for the BD FACSPresto study were not included in this study. The study participants were categorized into two groups—HIV-negative and HIV-positive groups based on the presence of HIV infection. The HIV-negative group comprised of individuals with no previous history of HIV infection or AIDS, confirmed by a negative HIV antibody test. This group included, healthy subjects and patients with preexisting diseases such as, hypertension, diabetes, and hyperlipidemia. Healthy subjects were defined as those without any preexisting clinical diseases and having normal hematologic indexes; a routine blood examination during the previous 7 days showed white blood cell counts, differential counts, and Hb levels to be within the normal reference ranges.

This study was approved by the ethics committees of the Beijing YouAn Hospital of the Capital Medical University, the Shanghai Tongren Hospital, and the Shanghai Public Health Clinical Center. Written informed consent was obtained from all subjects prior to enrollment. All procedures performed in studies involving human participants were in accordance with the ethical standards of the institutional and/or national research committee and with the 1964 Helsinki declaration and its later amendments or comparable ethical standards.

### Sample collection and storage

Simultaneous venous and finger-prick (capillary) blood samples were taken by—9 study nurses, experienced in finger-prick and venous blood sampling. Capillary blood sampling was performed in accordance with the document issued by the Clinical Laboratory Standards Institute (CLSI) (GP42-A6)^25^. Venous blood sample was collected as part of routine laboratory monitoring for all HIV-positive patients by trained laboratory staff. Briefly, 1 mL of venous blood was collected in a blood collection tube containing EDTA anticoagulant and stored at room temperature (20–25 °C); while the blood smear sample stained with monoclonal antibodies was analyzed within 24 h.

Venous and capillary blood samples collected were tested on the BD FACSPresto analyzer and BD FACSCalibur flow cytometer (Becton Dickinson, San Jose California, USA) for CD4 counts and Sysmex XT-4000i hematology analyzer (Sysmex Corporation, Sysmex America, Inc USA) for Hb levels by trained and experienced laboratory personnel.

HIV-negative individuals (confirmed by HIV testing) were evaluated within a week of providing the written informed consents.

### Testing on the BD FACSPresto system for the measurement of AbsCD4, CD4%, and Hb concentration

Testing on the BD FACSPresto system (Becton Dickinson, San Jose California, USA) was performed for the enumeration of CD4 absolute count, CD4 percentage of lymphocytes, and determination of Hb concentration in normal and HIV-positive patients, in accordance with the manufacturer’s instructions. The BD FACSPresto CD4/Hb Cartridge (Catalog No. 660746), a single use reagent cartridge, was used with the BD FACSPresto System.

Briefly, the venous and finger-prick blood samples [capillary sample was collected aseptically from fingertip using 1.8 mm depth lancet finger stick (Becton Dickinson Biosciences, San Jose California, USA)] were collected directly onto the test strip and were transferred to the labeled BD FACSPresto CD4/Hb cartridges. The cartridge cap was then closed and placed onto the BD FACSPresto platform outside the instrument for about 18 min to allow incubation at room temperature. The BD FACSPresto CD4/Hb cartridge contains in-built control features to check the analyzer and reagent functionality daily and this ensured the reliability of the system. Following incubation, the test strip was removed and the cartridge was then inserted into the analyzer to interpret the result. Subsequently, the AbsCD4, CD4% results are displayed on the screen and printed automatically. The result was used for research purposes only and was neither handed over to the client nor used for patients’ clinical management.

### Reference standards

The BD Tritest CD3/CD4/CD45 assay kit [50 tests/kit; National Food and Drug Administration Instrument (Import) 2014 registration certificate No. 3401679], which is commercially available in China, was selected as the reference assay for the measurement of AbsCD4 and CD4% in venous blood. This kit comprises BD Tritest CD3 fluorescein isothiocyanate (FITC)/CD4 phycoerythrin (PE)/CD45 peridinin chlorophyll protein (PerCP), a three-color direct immunofluorescence reagent, which was used with the BD FACSCalibur flow cytometer (Becton Dickinson Biosciences, San Jose California, USA) for the enumeration of lymphocyte subset percentages and absolute counts in whole blood samples (Supplementary Fig. 1). The CD4 estimation using the BD FASCalibur system was carried out using a standard methodology as previously described^26^. Briefly, 50 µL of venous blood was dispensed into a BD Trucount tube, 20 µL of BD Tritest CD3/CD4/CD45 reagent was added, mixed and incubated for 15 min, followed by the addition of 450 µL of lysing solution and incubation for another 15 min. The stained samples were acquired in the BD FACSCalibur system using the CD3/CD4/CD45 application of the BD Multiset software.

The Sysmex XT-4000i automatic hematology analyzer [National Food and Drug Administration Instrument (Import) 2014 registration certificate No. 2400324; Sysmex, Kobe, Japan] was used as the reference standard for determining Hb concentration in venous blood samples.

### Statistical analyses

The data were analyzed using SAS software (version 9.4, SAS Institute, Cary, NC, USA). Categorical data are presented as *n*(%), and measurement data are presented as mean ± standard deviation.

Method comparison studies were performed using Deming regression and Bland–Altman plots. Deming regression was performed in which CD4 counts and CD4 percentage of lymphocytes from the test (BD FACSPresto system using venous blood and capillary blood samples) were compared to the reference method (BD FACSCalibur using venous blood samples) to determine the analytical accuracy. Deming regression analysis demonstrated both constant and proportional biases using intercept and slope parameters. Further, the regression coefficient (R^2^) between the BD FACSPresto, the BD FACSCalibur and/or Sysmex XT-4000i and 97.5% confidence interval (97.5% CI) of the slope were analyzed. The set criteria for acceptable performance of the BD FACSPresto system were R^2^ > 0.90 and a 97.5% CI of the slope with the range of 0.85–1.15.

The Bland–Altman (deviation analysis) plot was used to determine the mean bias and 95% limit of agreement (LOA = mean bias ± 1.96 standard deviation [SD] of the differences of the results obtained)^27^. The LOA is calculated by using the mean difference ± the SD of the difference times the multiplier value. Assuming a normal distribution, “1.96” is used as the multiplier value as 95% of the data fall within these limits^28,29^. For this, the difference between the two methods of measurement was graphically represented on the Y-axis against their mean on the X-axis. The absolute deviation was calculated as: value obtained using the BD FACSPresto system—value obtained by the reference standard. The relative deviation or percentage similarity was calculated as: (absolute deviation/value obtained by the reference standard) × 100^30^. The primary endpoints were comparisons of the measurements of AbsCD4 in venous and capillary blood samples generated by the BD FACSPresto system with that of those in venous blood obtained by using the reference method. The secondary endpoints were comparisons of the measurements of CD4% and Hb in venous and capillary blood samples using the BD FACSPresto system with that of those in venous blood obtained by using the reference method. All statistical tests were two-sided, and *P* < 0.05 was considered statistically significant.

## Results

### Enrollment, characteristics and analyses sets of the study participants

All experiments were performed in triplicate (Supplementary Figs. 2, 3, and 4). A total of 1304 (N = 1304) subjects were enrolled in this study. Of these, some samples from the protocol set were excluded from our analyses due to instrument issues or not processed as per protocol (poor sample quality, process controls were not tested prior to sample testing, samples were processed outside the manufacturer’s recommended time window for sample staining and/or acquisition, acquisition did not satisfy the minimum number required of lymph events, incorrect storage conditions of the venous blood sample, where the samples were not properly stored in EDTA at 20–25 °C up to 24 h after collection, and reagent storage issues). Specimens with only valid results were analyzed. Results were considered invalid if testing did not comply with the protocol procedures (such as testing outside the time window) or if system errors (QC failure, non-reads) suppressed results.

During acquisition, if the instrument displayed an error code, the laboratory personnel typically re-ran the cartridges. A cartridge failure implied an unsuccessful attempt to generate results after a repeated acquisition of the same cartridge. Therefore, we re-prepared using a new cartridge.

Participant enrollment flowcharts including, exclusion criteria for the measurements of AbsCD4, CD4% and Hb concentrations in both venous and capillary blood samples are shown in Supplementary Figs. 2, 3, and 4, respectively. Following exclusions, a total of 1033 (n = 1033) and 1016 (n = 1016) samples were included in the final analyses sets for comparing the measurements of AbsCD4 generated by the BD FACSPresto system in venous and capillary blood samples, respectively, with that of those in venous blood obtained by using the BD FACSPresto system. Similarly, a total of 1025 (n = 1025) and 1009 (n = 1009) samples were included in the final analyses sets for comparing the measurements of CD4% generated by the BD FACSPresto system in venous and capillary blood samples, respectively, with that of those in venous blood obtained by using the BD FACSPresto system. Further, a total of 1069 (n = 1069) and 1048 (n = 1048) samples were included in the final analyses sets for comparing the measurements of total hemoglobin concentrations generated by the BD FACSPresto system in venous and capillary blood samples, respectively, with that of those in venous blood obtained by using the Sysmex XT-4000i analyzer.

### System equivalency and method comparisons

All venous blood samples had a corresponding capillary sample from the same individual. All samples were tested on the FACSPresto and the FACSCalibur systems. The accuracy and precision of the BD FACSPresto system were assessed by comparing the absolute CD4 count and CD4 percentage results against those obtained from the BD FACSCalibur system (Reference standard). For Hb, we compared the results obtained from the Sysmex XT-4000i hematology analyzer (venous blood samples) with those obtained from the BD FACSPresto system (venous and capillary blood samples). Our valid test results were well within the reference ranges, as specified in the manufacturer’s instructions. For BD FACSPresto system (venous and fingertip capillary blood samples): AbsCD4: 462–1306 cells/μL (female) and 440–1602 cells/μL (male); CD4%: 32–55% (female) and 29–54% (male). For BD FACSCalibur (venous blood samples): CD4 count: 410–1590 cells/μL; CD4%: 31–60%; For BD FACSPresto system (venous and fingertip capillary blood samples): hemoglobin concentration: 120–160 g/L (female) and 135–180 g/L (male). And for Sysmex XT-4000i analyzer (venous blood samples): hemoglobin concentration: 115–152 g/L (female) and 133–175 g/L (male).

#### Performance comparison between the BD FACSPresto system and the FACSCalibur flow cytometer in the measurement of AbsCD4

**Figure 1:**
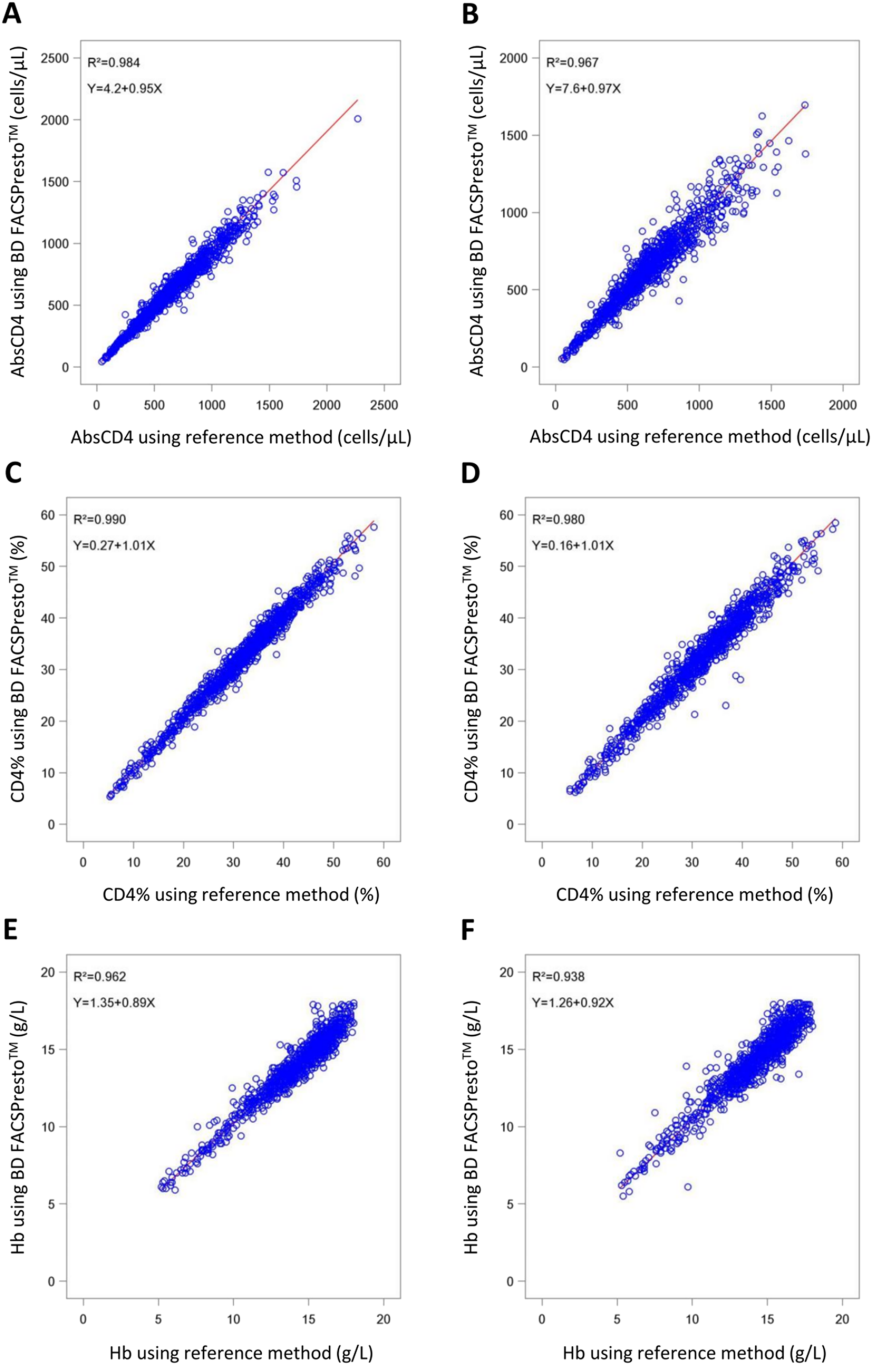
Deming regression analyses (all study participants). (**A**) Correlation between absolute CD4 cell count (AbsCD4) in venous blood measured using the BD FACSPresto system and AbsCD4 in venous blood measured using the reference standard (BD Tritest kit and BD FACSCalibur system). (**B**) Correlation between AbsCD4 in capillary blood measured using the BD FACSPresto system and AbsCD4 in venous blood measured using the reference standard (BD Tritest kit and BD FACSCalibur system). (**C**) Correlation between CD4 T lymphocyte percentage of all lymphocytes (CD4%) in venous blood measured using the BD FACSPresto system and CD4% in venous blood measured using the reference standard (BD Tritest kit and BD FACSCalibur system). (**D**) Correlation between CD4% in capillary blood measured using the BD FACSPresto system and CD4% in venous blood measured using the reference standard (BD Tritest kit and BD FACSCalibur system). (**E**) Correlation between hemoglobin concentration (Hb) in venous blood measured using the BD FACSPresto system and Hb in venous blood measured using the reference standard (Sysmex XT-4000i analyzer). (**F**) Correlation between Hb in capillary blood measured using the BD FACSPresto system and Hb in venous blood measured using the reference standard (Sysmex XT-4000i analyzer).

**Figure 2:**
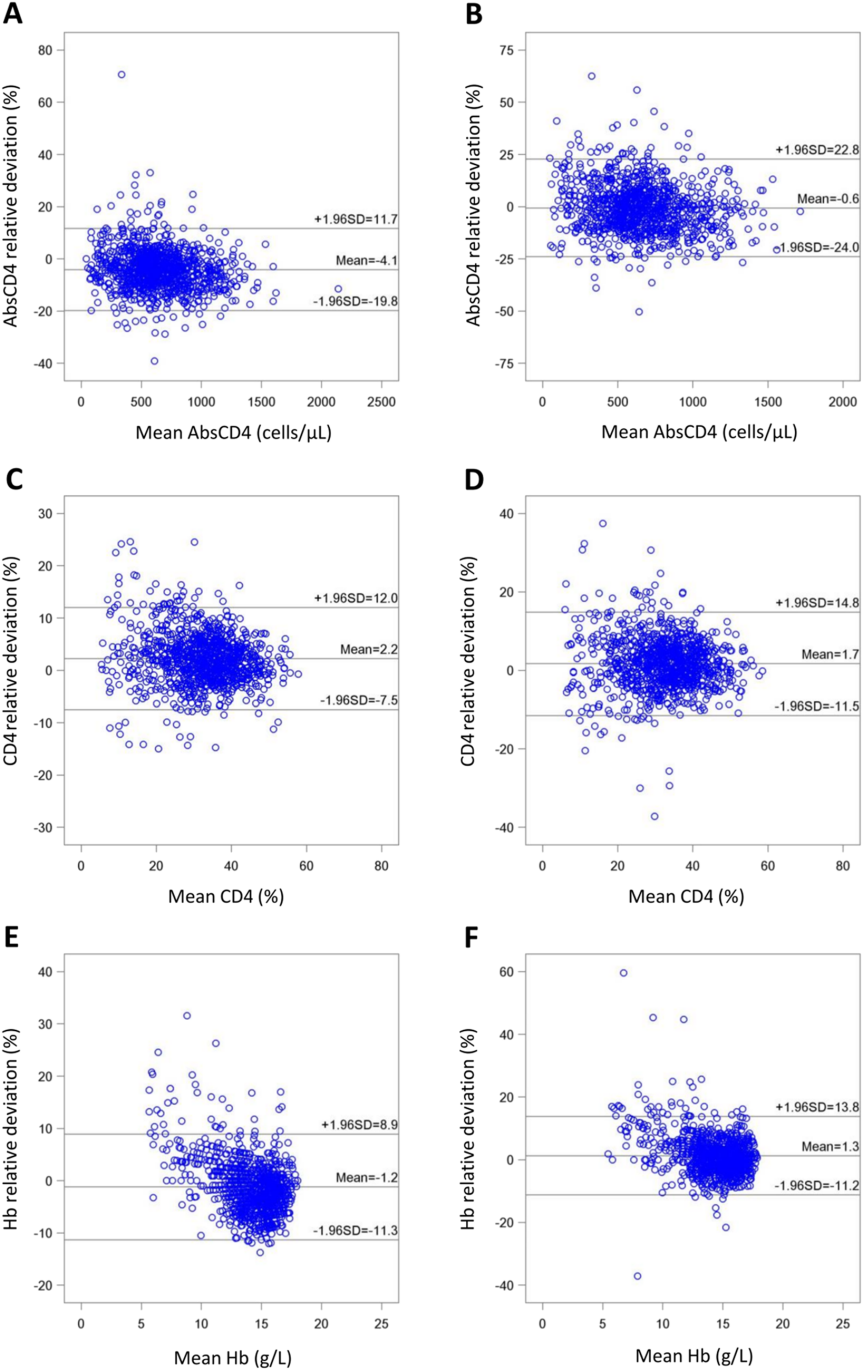
Bland–Altman analyses of relative deviations (all study participants). (**A**) Deviation analysis of absolute CD4 cell count (AbsCD4) in venous blood measured using the BD FACSPresto system and AbsCD4 in venous blood measured using the reference standard (BD Tritest kit and BD FACSCalibur system). (**B**) Deviation analysis of AbsCD4 in capillary blood measured using the BD FACSPresto system and AbsCD4 in venous blood measured using the reference standard (BD Tritest kit and BD FACSCalibur system). (**C**) Deviation analysis of CD4 T lymphocyte percentage of all lymphocytes (CD4%) in venous blood measured using the BD FACSPresto system and CD4% in venous blood measured using the reference standard (BD Tritest kit and BD FACSCalibur system). (**D**) Deviation analysis of CD4% in capillary blood measured using the BD FACSPresto system and CD4% in venous blood measured using the reference standard (BD Tritest kit and BD FACSCalibur system). (**E**) Deviation analysis of hemoglobin concentration (Hb) in venous blood measured using the BD FACSPresto system and Hb in venous blood measured using the reference standard (Sysmex XT-4000i analyzer). (**F**) Deviation analysis of Hb in capillary blood measured using the BD FACSPresto system and Hb in venous blood measured using the reference standard (Sysmex XT-4000i analyzer).

Deming regression analysis (Fig. 1) comparing the accuracies of the BD FACSPresto system and the reference standard for the measurement of AbsCD4 in venous blood samples FACSPresto demonstrated a significant linear correlation (*P* < 0.001) between the CD4 values generated by the two platforms, with slope values of 0.95, 0.96 and 0.94 and R^2^ values of 0.984, 0.982 and 0.960, for all participants, and for those in the HIV-positive, and the HIV-negative groups, respectively (Table 1). Bland–Altman plots (Fig. 2) revealed that the mean relative deviations or mean %biases with 95% LOA for CD4 cells were − 4.05%, − 2.99% and − 4.76% for all participants, and for those in the HIV-positive, and the HIV-negative groups, respectively, and approximately ≥ 95% of participants in each of these three groups were within the mean ± 1.96 SD of the relative deviation (Table 1).

**Table 1:** Summary of Deming regression and Bland–Altman analyses comparing the measurements of absolute CD4 cell counts (AbsCD4) between the BD FACSPresto system and the reference standard (BD Tritest kit and BD FACSCalibur system).

	BD FACSPresto using venous blood sample vs BD Tritest/FACSCalibur using venous blood sample			BD FACSPresto using capillary blood sample vs BD Tritest/FACSCalibur using venous blood sample		
	All *N* = 1033	HIV-positive group *N* = 402	HIV-negative group *N* = 631	All *N* = 1016	HIV-positive group *N* = 390	HIV-negative group *N* = 626
**Deming regression**						
Intercept	4.2	2.3	8.3	7.6	5.1	16.0
Slope	0.95	0.96	0.94	0.97	0.99	0.95
97.5% CI of slope	0.94–0.96	0.95–0.97	0.92–0.95	0.96–0.99	0.96–1.01	0.93–0.98
R^2^	0.984	0.982	0.960	0.967	0.965	0.909
*P* value	< 0.001*	< 0.001*	< 0.001*	< 0.001*	< 0.001*	< 0.001*
**Absolute deviation**						
Mean ± SD	− 32.4 ± 57.9	− 19.9 ± 47.9	− 40.4 ± 62.2	− 13.5 ± 82.8	− 3.0 ± 69.2	− 20.1 ± 89.7
95% CI	− 35.9 to − 28.9	− 24.6 to − 15.2	− 45.2 to − 35.5	− 18.6 to − 8.4	− 9.9 to 3.9	− 27.1 to − 13.0
Within mean ± 1.96 SD	970 (93.9%)	381 (94.8%)	592 (93.8%)	961 (94.6%)	364 (93.3%)	593 (94.7%)
**Relative deviation**						
Mean ± SD	− 4.05 ± 8.02	− 2.99 ± 8.97	− 4.76 ± 7.29	− 0.62 ± 11.95	0.92 ± 12.85	− 1.58 ± 11.25
95% CI	− 4.54 to − 3.56	− 3.87 to − 2.11	− 5.29 to − 4.16	− 1.35 to 0.12	− 0.36 to 2.20	− 2.46 to − 0.69
Within mean ± 1.96 SD	988 (95.6%)	382 (95.0%)	601 (95.2%)	967 (95.2%)	370 (94.9%)	594 (94.9%)

*CI* confidence interval, *SD* standard deviation.

*P value < 0.05 is considered significant.

Strong positive correlations were also observed between the AbsCD4 values generated by the BD FACSPresto system (using capillary blood samples) and the reference standard technology (using venous blood samples) (Fig. 1). The corresponding Deming regression results showed slope values of 0.97, 0.99, and 0.95 (*P* < 0.001) and R^2^ values of 0.967, 0.965, and 0.909, respectively, for all participants, and for those in the HIV-positive, and the HIV-negative groups (Table 1). Furthermore, the mean %biases with 95% LOA for all participants, and for those in the HIV-positive, and the HIV-negative groups were − 0.62%, 0.92%, and − 1.58%, respectively, and approximately ≥ 94.9% of participants in each group were within the mean ± 1.96 SD of the relative deviation (Table 1; Fig. 2).

#### Performance comparison between the BD FACSPresto system and the FACSCalibur flow cytometer in the measurement of CD4%

Deming regression analysis (Fig. 1) demonstrated significant linear correlations (*P* < 0.001) between measurements of CD4% generated by the BD FACSPresto system and the reference standard technology (using venous blood samples), with slope values of 1.01, 1.01, and 1.00 and R^2^ values of 0.990, 0.986, and 0.964, respectively, for all participants, and for those in the HIV-positive, and the HIV-negative groups (Table 2). Bland–Altman plots (Fig. 2) yielded mean relative deviations of 2.25%, 2.32%, and 2.20%, respectively, for all participants, and for those in the HIV-positive, and the HIV-negative groups, and approximately ≥ 93.2% of participants in each of these groups were within the mean ± 1.96 SD of the relative deviation (Table 2).

**Table 2:** Summary of Deming regression and Bland–Altman analyses comparing the CD4 percentage values (CD4%) between the BD FACSPresto system and the reference standard (BD Tritest kit and BD FACSCalibur system).

	BD FACSPresto using venous blood sample vs BD Tritest/FACSCalibur using venous blood sample			BD FACSPresto using capillary blood sample vs BD Tritest/FACSCalibur using venous blood sample		
	All *N* = 1025	HIV-positive group *N* = 397	HIV-negative group *N* = 628	All *N* = 1009	HIV-positive group *N* = 385	HIV-negative group *N* = 624
**Deming regression (linear regression)**						
Intercept	0.27	0.33	0.62	0.16	0.34	0.60
Slope	1.01	1.01	1.00	1.01	0.99	1.00
97.5% CI of slope	1.00–1.02	0.99–1.02	0.99–1.02	1.00–1.02	0.98–1.01	0.98–1.02
R^2^	0.990	0.986	0.964	0.980	0.972	0.929
*P* value	< 0.001*	< 0.001*	< 0.001*	< 0.001*	< 0.001*	< 0.001*
**Absolute deviation**						
Mean ± SD	0.61 ± 1.41	0.41 ± 1.30	0.73 ± 1.46	0.43 ± 1.96	0.13 ± 1.83	0.62 ± 2.02
95% CI	0.52 to 0.70	0.29 to 0.54	0.62 to 0.85	0.31 to 0.55	− 0.05 to 0.32	0.46 to 0.78
Within mean ± 1.96 SD	980 (95.6%)	377 (95.0%)	602 (95.9%)	961 (95.2%)	366 (95.1%)	600 (96.2%)
**Relative deviation**						
Mean ± SD	2.25 ± 4.97	2.32 ± 5.97	2.20 ± 4.23	1.68 ± 6.72	1.25 ± 7.95	1.94 ± 5.82
95% CI	1.94–2.55	1.73–2.91	1.87–2.53	1.26–2.09	0.45–2.04	1.48–2.40
Within mean ± 1.96 SD	966 (94.2%)	370 (93.2%)	596 (94.9%)	963 (95.4%)	366 (95.1%)	600 (96.2%)

*CI* confidence interval, *SD* standard deviation.

*P value < 0.05 is considered significant.

Further, significant correlations were observed between the CD4% values obtained from the BD FACSPresto system (using capillary blood samples) and the reference standard (*P* < 0.001), where slope values were 1.01, 0.99, and 1.00 and R^2^ values of 0.980, 0.972, and 0.929 for all participants, and for those in the HIV-positive, and the HIV-negative groups, respectively (Table 2; Fig. 1). The mean relative deviations for all participants, and for those in the HIV-positive, and the HIV-negative groups were 1.68%, 1.25%, and 1.94%, respectively, and approximately ≥ 95.1% of participants in each group were within the mean ± 1.96 SD of the relative deviation (Table 2; Fig. 2).

#### Performance comparison between the BD FACSPresto system and Sysmex XT-4000i automatic hematology analyzer for the measurement of Hb concentrations

For Hb concentrations in venous blood samples, we observed significant correlations (*P* < 0.001) between the values generated by the BD FACSPresto system and the reference standard, where slope values were 0.89, 0.86, and 0.91 and R^2^ values of 0.963, 0.953, and 0.969 for all participants, and for those in the HIV-positive, and the HIV-negative groups, respectively (Table 3). In addition, Bland–Altman plots (Fig. 2) yielded mean relative deviations of − 1.19%, − 2.57%, and − 0.28% for all participants, and for those in the HIV-positive, and the HIV-negative groups, respectively, and approximately ≥ 95.8% of participants in each of these groups were within the mean ± 1.96 SD of the relative deviation (Table 3).

**Table 3:** Summary of Deming regression and Bland–Altman analyses comparing the hemoglobin concentration values between the BD FACSPresto system and the reference standard (Sysmex XT-4000i analyzer).

	BD FACSPresto using venous blood sample vs Sysmex XT-4000i using venous blood sample			BD FACSPresto using capillary blood sample vs Sysmex XT-4000i using capillary blood sample			
	All *N* = 1069	HIV-positive group *N* = 426	HIV-negative group *N* = 643	All *N* = 1048		HIV-positive group *N* = 416	HIV-negative group *N* = 632
**Deming regression (linear regression)**							
Intercept	1.35	1.69		1.08	1.26	1.12	1.23
Slope	0.89	0.86		0.91	0.92	0.92	0.93
97.5% CI of slope	0.87–0.90	0.83–0.88		0.90–0.93	0.90–0.94	0.87–0.97	0.90–0.95
R^2^	0.962	0.953		0.969	0.938	0.917	0.951
*P* value	< *0.001*	< *0.001*		< *0.001*	< *0.001*	< *0.001*	< *0.001*
**Absolute deviation**							
Mean ± SD	− 0.24 ± 0.66	− 0.46 ± 0.72		− 0.09 ± 0.58	0.11 ± 0.77	− 0.07 ± 0.87	0.23 ± 0.68
95%CI	− 0.28 to − 0.20	− 0.53 to − 0.39		− 0.14 to − 0.05	0.06 to 0.16	− 0.16 to 0.01	0.18 to 0.28
Within mean ± 1.96 SD	1016 (95.0%)	408 (95.8%)		609 (94.7%)	1002 (95.6%)	398 (95.7%)	597 (94.5%)
**Relative deviation**							
Mean ± SD	− 1.19 ± 5.15	− 2.57 ± 5.57		− 0.28 ± 4.64	1.31 ± 6.38	0.02 ± 6.74	2.18 ± 5.98
95% CI	− 1.50 to − 0.88	− 3.10 to − 2.04		− 0.64 to 0.08	0.92 to 1.69	− 0.67 to 0.63	1.71 to 2.65
Within mean ± 1.96 SD	1024 (95.8%)	410 (96.2%)		616 (95.8%)	1003 (95.7%)	394 (94.7%)	606 (95.9%)

*CI* confidence interval, *SD* standard deviation.

*P value < 0.05 is considered significant.

Notably, Hb concentration values in capillary blood samples measured using the BD FACSPresto system strongly correlated with those obtained from the reference standard technology (*P* < 0.001), where slope values were 0.92, 0.92, and 0.93 and R^2^ values of 0.938, 0.917 and 0.951, respectively for all participants, and for those in the HIV-positive, and the HIV-negative groups (Table 3; Fig. 1). Expectedly, the mean %biases for all participants, and for those in the HIV-positive, and the HIV-negative groups were 1.31%, 0.02%, and 2.18%, respectively, and approximately ≥ 94.7% of participants in each of the three groups were within the mean ± 1.96 SD of the relative deviation (Table 3; Fig. 2).

## Discussion

Our study is the first-of-its-kind to validate the BD FACSPresto system for the measurement of CD4 T-lymphocytes and total hemoglobin concentration in venous and capillary blood samples of Chinese patients with HIV infection. Our findings showed an acceptable agreement between the BD FACSPresto and the BD FACSCalibur systems for CD4 T-cell counting and CD4%; and between the BD FACSPresto and Sysmex XT-4000ifor measuring Hb concentration in venous and capillary blood samples. Further, we demonstrated that the BD FACSPresto system was accurately able to quantify CD4 T cell count, CD4%, and Hb concentration using capillary and venous blood from adult non-HIV and HIV-infected individuals in China. The portability of the BD FACSPresto system and its ability to accurately measure three critical parameters (AbsCD4, CD4%, and Hb levels) that reflect the clinical status of people living with HIV/AIDS makes it well-suited for use as a point-of-care CD4 testing technology. Assuming that the BD FACSPresto system has been successfully validated in a real-world setting, we anticipate that widespread implementation of the BD FACSPresto system in China could dramatically improve monitoring of patients living with HIV/AIDS, particularly in resource-limited regions of the country. Notably, our findings are supported by the hypothesis—the results for AbsCD4, %CD4, and Hb concentration generated by the BD FACSPresto system are accurate and reproducible when samples are measured within 24 h of phlebotomy^23^. Collectively, these indicate the accuracy and reproducibility of the BD FACSPresto technology. Lending further strength to our results, a multicentric study by Thakar et al.^23^, which included HIV-positive patients from Kenya, India, Thailand, and the USA validated the accuracy, precision, and reliability of the BD FACSPresto system and demonstrated a good agreement with gold-standard methods, the BD Tritest CD3/CD4/CD45 reagent/BD FACSCalibur system (for measurements of AbsCD4 and CD4%) and a Sysmex hematology analyzer (for determining Hb concentration), irrespective of venous or capillary blood sampling. In addition, the Deming regression results for CD4 counts and %CD4 gave slopes within 0.96–1.05 and R^2^ ≥ 0.96; Hb slopes were ≥ 1.00 and R^2^ ≥ 0.89, similar to the findings of this study. Further strengthening the results of our study, another study by Angira et al. assessed the performance of the BD FACSPresto system and further evaluated its accuracy, stability, linearity, precision, and reference intervals using capillary and venous blood of HIV-positive patients from Kenya; the Deming regression slopes for AbsCD4 and CD4% (venous/capillary) were within 0.97–1.03 and R^2^ was ≥ 0.96; while slope and R^2^ values were ≥ 0.94 for Hb^26^. Furthermore, in line with several previously published studies^23,26,31,32^, Bland–Altman analyses demonstrated close agreement between the BD FACSPresto system and the reference standards for all study participants. A notable advantage of the BD FACSPresto system is that testing can be performed using the finger-prick (capillary) blood sample without compromising the accuracy of analytical results, thus simplifying and improving the efficiency of diagnostics for HIV/AIDS. Corroborating the results of this study, previous studies have shown equivalency of CD4 cell count between venous and capillary blood^33,34^.

The Chinese government has provided free HIV testing and free ART since 2003^35^, although suboptimal clinical outcomes remain a problem^36^. The use of the BD FACSPresto system for POC testing of AbsCD4 and CD4% potentially could improve the viral load monitoring of people living with HIV in China, particularly in rural areas and resource-limited settings. CD4 T cell testing is an important diagnostic tool that offers valuable insights of the immune system status for monitoring and long-term care management, disease stage and progression, risk of opportunistic infections and mortality risk^37,38^; therefore, enumerating absolute CD4 counts is key to prioritizing decisions related to the initiation of ART in settings where standard treatment is unavailable^39^. Furthermore, although viral load is considered superior to CD4 cell count for monitoring the response to ART^37^, CD4 cell count remains the single most important parameter in places where viral load testing is not available^8–10^. Increasing the availability of POC testing at several sites could potentially impact treatment outcomes, as higher patient load especially, at village clinics and travel distance are associated with increased risk of HIV transmission^40^.

Anemia is a common hematologic abnormality in people living with HIV in China^13,14^. Given the lower CD4 cell counts and higher viral load, anemia has been shown to be associated with worse outcomes^15,16,41^. Furthermore, anemia is a recognized adverse effect of zidovudine-containing treatment regimens among patients initiated with ART^20,21^. A notable feature of the BD FACSPresto system is that it simultaneously identifies and enumerates CD4 T lymphocytes for absolute and percentage results and further measures total hemoglobin concentration on the same sample and delivers all results concurrently. The BD FACSPresto system could therefore, simplify anemia detection in patients receiving ART combination therapy that includes ZDV, thereby facilitating quick decisions regarding any changes in the treatment strategy.

This study has several limitations that should be taken into consideration when interpreting the results. The study participants were enrolled from only three centers in two major cities of China. Furthermore, the collection of venous and capillary blood samples and the measurement of AbsCD4, CD4% and Hb level using the BD FACSPresto system were performed by fully-trained medical staff in a central laboratory facility. Of note, the study participants were enrolled using strict inclusion and exclusion criteria, where patients were excluded even if there were problems with acquisition or processing of blood samples. Therefore, this necessitates further research to validate the BD FACSPresto system in a real-world setting and establish whether our findings are generalizable to other scenarios, especially in resource-constrained clinics in rural regions, where POC systems are often operated by non-laboratory trained personnel.

## Conclusions

This study is the first-of-its-kind to validate the accuracy of BD FACSPresto system in Chinese population. The BD FACSPresto system displayed equivalent performance compared with the standard-of-care systems for the measurement of AbsCD4, CD4%, and Hb levels in venous and capillary blood samples of HIV-infected patients. Most importantly, the BD FACSPresto system simultaneously measured all the three key parameters rapidly and accurately thus, simplifying the process of monitoring patients with HIV in a single visit. Furthermore, the interface of the BD FACSPresto system is straightforward, relatively easy to use, and requires only basic training for its operation and preparation of blood samples. Therefore, the BD FACSPresto system is well-suited for use in POC testing at remote facilities where resources are limited, which would in turn improve access to patients living in remote areas. This is particularly relevant given the fact that POC improves linkage to HIV care and facilitates timely initiation of therapeutic intervention^42^. Should the BD FACSPresto system be validated in a real-world clinical setting, we anticipate that its widespread implementation in China could enhance the monitoring of patients living with HIV/AIDS, especially in resource-constrained regions of the country.

## Supplementary information

Supplementary Figures.

## Data Availability

The datasets used and/or analyzed during the current study are available from the corresponding author on reasonable request.
