# Supplementary material for: Validation of the BD FACSPresto system for the measurement of CD4 T-lymphocytes and hemoglobin concentration in HIV-negative and HIV-positive subjects
Source: Sci Rep. 2020 Nov 11;10:19605. doi: 10.1038/s41598-020-76549-6 (PMC7658244; doi:10.1038/s41598-020-76549-6)
Supplement: Supplementary file 1 — Supplementary Figures. [file 41598_2020_76549_MOESM1_ESM.doc]

**Validation of the BD FACSPresto system for the measurement of CD4 T-lymphocytes and hemoglobin concentration in HIV-negative and HIV-positive subjects**

Xiaofan Lu^1, 2¶^, Hanxiao Sun^3¶^, Haicong Li^4¶^, Wei Xia^1^, Hao Wu^1, 2^, Daihong Chen^4^, Meiyu Tan^3^, Shijun Yu^5^, Tong Zhang^1, 2*^, Huiming Sheng^3*^, Zhaoqin Zhu^4*^

^1^Center for Infectious Diseases, Beijing Youan Hospital, Capital Medical University, Beijing, China

^2^Beijing Key Laboratory for HIV/AIDS Research, Beijing, China

^3^Shanghai Tong Ren Hospital, Shanghai Jiaotong University School of Medicine, Shanghai, China

^4^Shanghai Public Health Clinical Center, Shanghai, China

^5^BD Biosciences, 2350 Qume Drive, San Jose, CA 95131, USA

^¶^These authors contributed equally to this work.

**^*^Corresponding author:**

Tong Zhang

^1^Center for Infectious Diseases, Beijing Youan Hospital, Capital Medical University, Beijing, China; ^2^Beijing Key Laboratory for HIV/AIDS Research, Beijing, China

E-mail: zt_doc@ccmu.edu.cn

Huiming Sheng

Shanghai Tong Ren Hospital, Shanghai Jiaotong University School of Medicine, Shanghai, China

Tel: +86-18917181019; E-mail: hmsheng@shsmu.edu.cn

Zhaoqin Zhu

Shanghai Public Health Clinical Center, Shanghai, China

E-mail: zhaoqinzhu@163.com

**Supplementary Figure 1. Flow cytometry data for the reference method.** (A) CD45/side scatter (SSC) plot with all events displayed. All CD45^+^ leukocytes are shown along with red blood cells, debris and standard flow-count beads. The data were used to set the threshold for CD45^+^ cells. R1 was set as the gate for lymphocytes. (B) CD3/CD4 plot with all events displayed. All CD45^+^ leukocytes are shown along with red blood cells, debris and standard flow-count beads. R2 was set as the gate for flow-count beads. (C) CD3/CD4 plot for gates R1 and R2, including lymphocytes and standard flow-count beads. R1 gates lymphocytes and R2 gates the standard flow-count beads. R3 was set for CD3^+^CD4^+^ cells, and R4 was set for CD3^+^CD4^-^ cells. (D) CD3/ SSC plot with all events displayed. All CD45^+^ leukocytes are shown along with red blood cells, debris and standard flow-count beads. R5 was set under R1 (lymphocytes) for CD3^+^ cells.


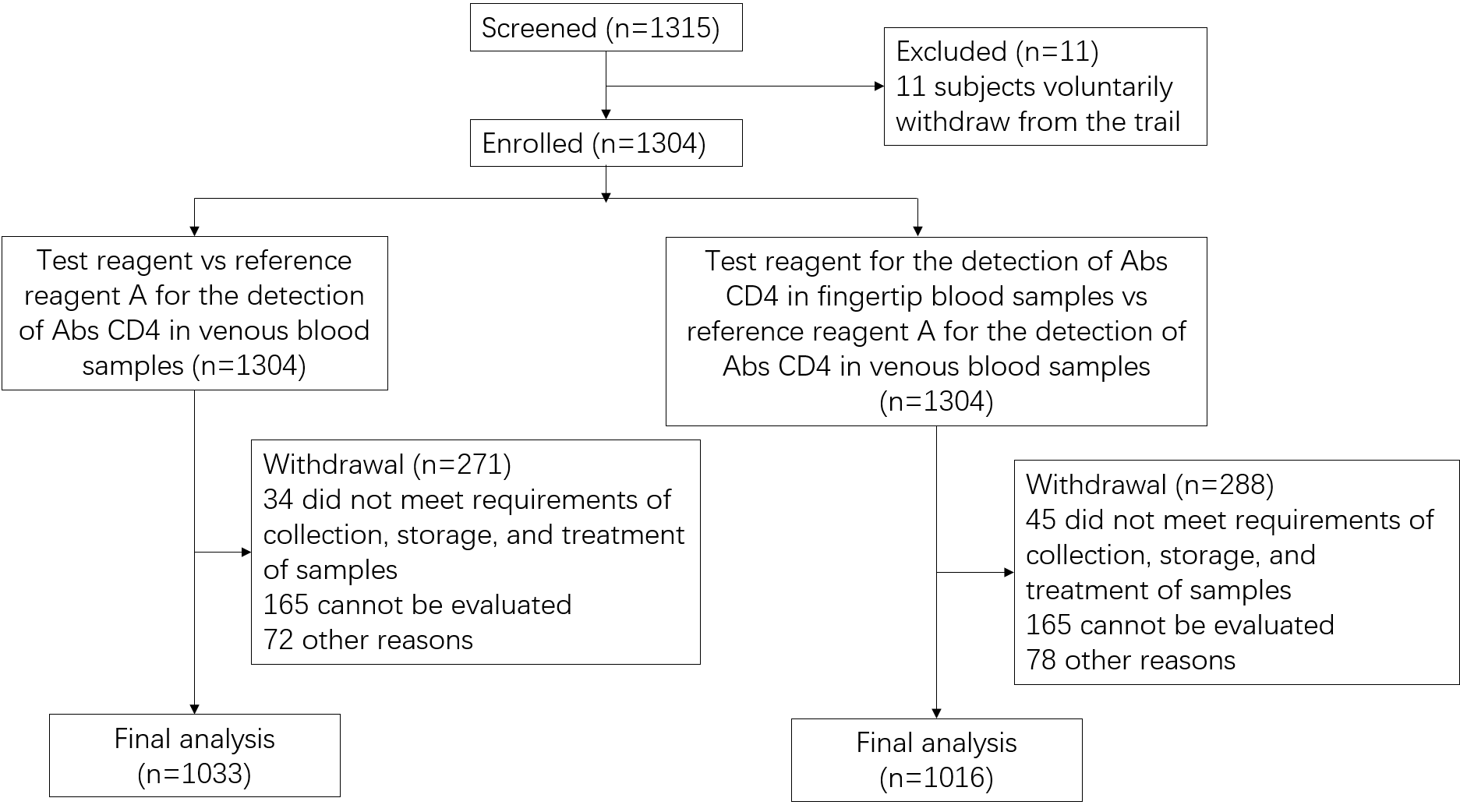


**Supplementary Figure 2.** **Participant enrollment flowchart showing exclusion criteria for the measurement of absolute CD4 cell counts (AbsCD4) using the BD FACSPresto system and the BD Tritest CD3/CD4/CD45 assay kit/BD FACSCalibur system.**


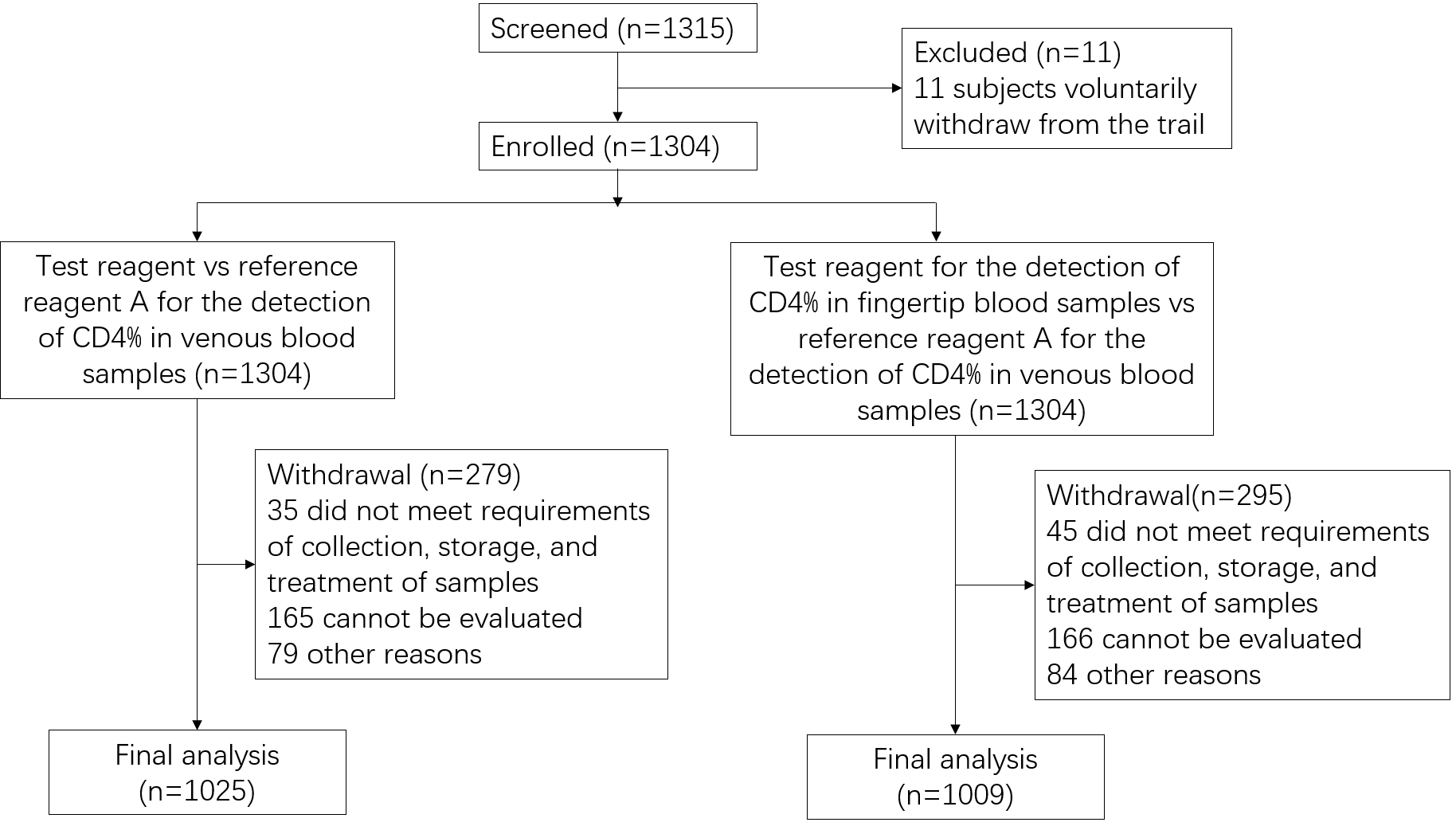


**Supplementary Figure 3.** **Participant enrollment flowchart showing exclusion criteria for the measurement of CD4 percentages of all lymphocytes (CD4%) using the BD FACSPresto system and the BD Tritest CD3/CD4/CD45 assay kit/BD FACSCalibur system.**


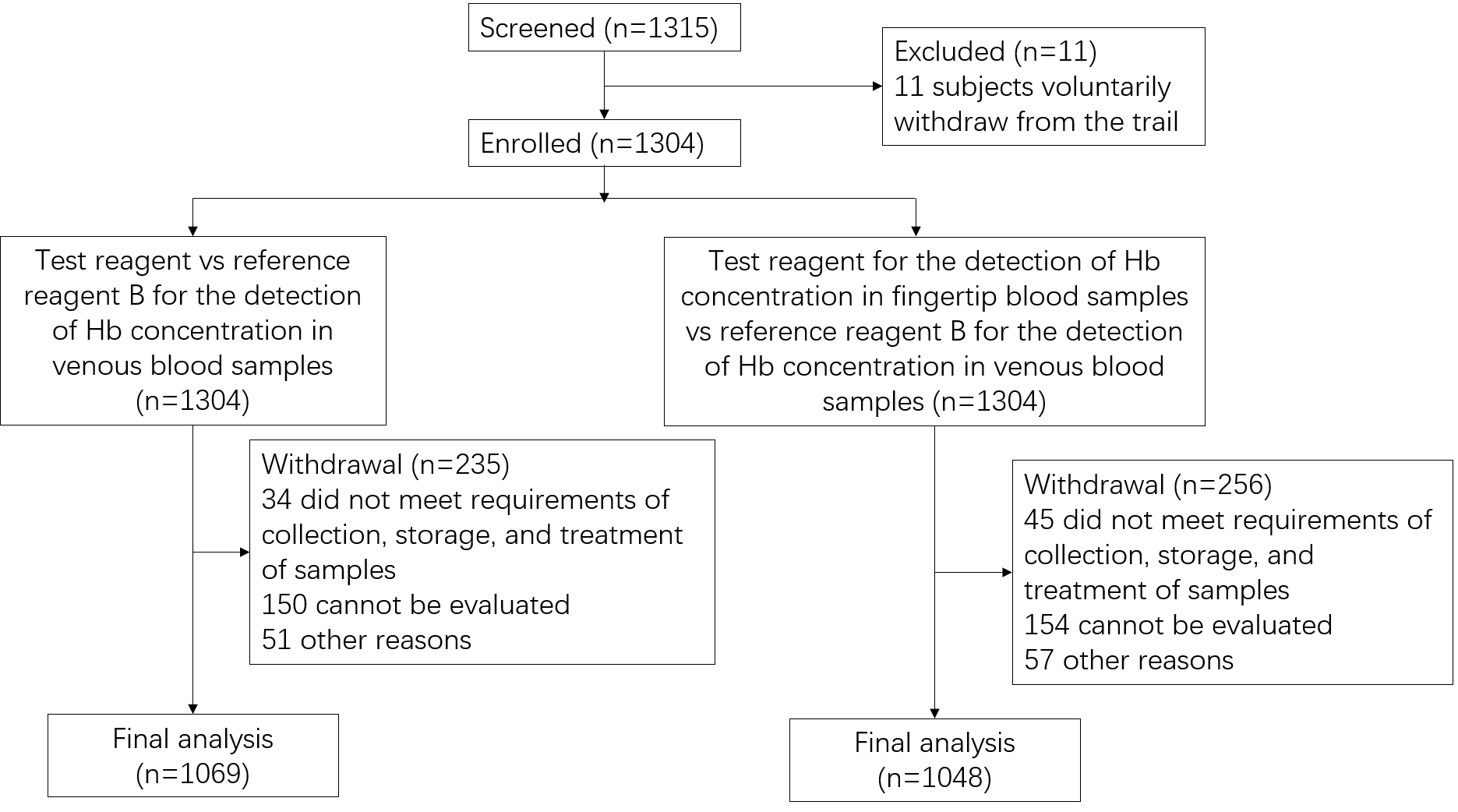


**Supplementary Figure 4.** **Participant enrollment flowchart showing exclusion criteria for the measurement of hemoglobin concentration using the BD FACSPresto system and the Sysmex XT-4000i automatic hematology analyzer.**
